# Quid novi in the eldery patient’s anesthesia

**DOI:** 10.1186/1471-2318-11-S1-A26

**Published:** 2011-08-24

**Authors:** B Lettieri, ML Mingione, A d’Elia, P Capodanno

**Affiliations:** 1 Department of Anaesthesia, Surgical and Emergency Science, Second University of Naples, Italy

## Background

Today the availability of new local anesthetics and the use of analgesics, allow the modulation of the analgesia, maintaining a state of consciousness.

**Figure 1:**
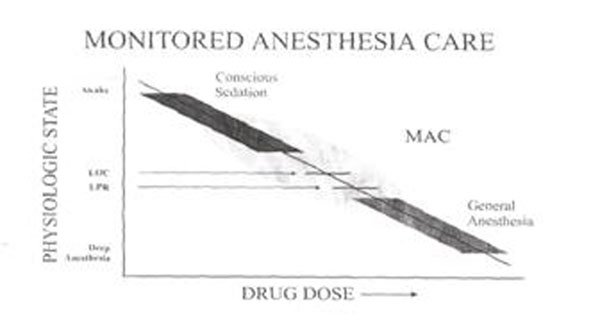
Monitored anestesia care

An answer to the needs of patients >75 years undergoing surgery is the technique Monitored Anesthesia Care (MAC), defined “the middle land” (Figure 1).

MAC allows:

- the modulation of the level of analgesia at different stages of surgery due to the availability of analgesic action, but with rapid onset-time

- the additional analgesia using local anesthetics with prolonged effect without the use of noradrenaline, dangerous for elderly patients

the consciousness and cooperation of the patient (Table 1).

**Table 1:** MAC.

***Conscious Sedation*** **(*****MAC*****)**	***Unconscious Sedation***
Altered consciousness	Unconsciousness
Conscious patient	Unconscious patient
Protective reflexes intact and active	Protective reflexes decreased; airway obstruction may occur Ventilation: hypoxia, hypercapnia Cardiovascular system: , hypotension, hypertension, bradycardia, tachycardia
Stable vital signs	Unstable vital signs
Analgesia may be present; need for regional analgesia / local or systemic	Pain controlled centrally; does not require regional analgesia
Limited stay in the units of observation	Requiring hospitalization or prolonged hospitalization
Low risk of complications	High risk of complications
Infrequent postoperative complications	Frequent postoperative complications
Patients with psychiatric problems or mental deficiency may be difficult to manage	May be needed to manage patients with mental deficiency

## Materials and methods

With this study we tested the efficacy, safety and limitations of the MAC.

The design of the study was a prospective, double-blind, parallel-group, with 42 patients randomly selected from 87 patients recruited between those eligible for inclusion in the circuit one-day surgery (Table 2)

**Table 2:** Patients’ criteria of homogeneity.

Patients’ criteria of homogeneity
same level of gravity ASA II/III
NYHA II class
same duration of surgery (40 min ± 10 min

Two groups were subjected to two different regimes of sedation with propofol and midazolam, pain controlled with remifentanil.

- Primary end-point was verifying the level and quality of sedation achieved

- Secondary end-point was identifying and quantifying potential adverse effects (Table 3-4)

**Table 3:** Access Criteria.

ACCESS CRITERIA
Weight 69 ± 6 Kg
Informed consent for MAC procedures
ASA II/III with stabilized cardio-circulatory impairments and respiratory parameters: pO_2_ ≤ 70 e pCO_2_ < 45 mmHg
Patients undergoing operations can be managed only with the cooperation of the patient
Age > 75 years

**Table 4:** Exclusion Criteria.

EXCLUSION CRITERIA
Patient desire
ASA III impairment of vital organs in acute and evolutionary phase
Patients with unexpected rapid intubation
Patients with high risk of bleeding
Severe neurological disorders

Levels of sedation, pain and mental status were assessed using different clinical approaches :

- Observational data (Table 5).

**Table 5:** Observer’ s assessment of alertness/sedation scale (oaa/s scale).

Answer	Verbal expression	Facial expression	Eyes	
Ready to the call, normal tone	Normal	Normal	Normal	5
Torpid to the call, normal tone	Initial slowdown	Medium relaxation	Medium relaxation	4
Only for repeat calls with high tone	slowdown	Marked relaxation	Marked ptosis	3
Only if shaken	Not understandable words	---	---	2
No answers, even if shaken	---	---	---	1

We proceeded as follows:

1) O_2_ inhalation (SpO_2_ > 98 and normocapnia)

2) during surgical manipulation a continuous infusion of remifentanil: 0.03 to 0.06 mg / kg / h was activated

Patients were randomly dichotomized into two arms with two different infusion regimens:

-group P (45 patients): starter bolus of 0.5 mg / kg propofol (to fill the central compartment) → P infusion of 1-2 mg / kg / h (to offset the rapid deployment)

-group M (41 patients): bolus starter from 0.03 to 0.05 mg / kg midazolam (average dose of 2-4 mg) infusion of 1-2 mg / kg / h

Every 10 m’ scores are recorded, BIS and OAA / S scale.

- objective parameters based on Ramsay Scale (Table 6).

**Table 6:** Ramsay Scale.

1	Patient anxious and agitated or restless, or both
2	Patient co-operative, orientated and tranquil
3	Patient responds to commands only
4	Brisk response to a light glabellar tap or auditory stimulus
5	Sluggish response to a light glabellar tap or auditory stimulus
6	No response to the stimuli mentioned in items 4 and 5

- Instrumental response with Bispectral Index (Table 7,8,9)

**Table 7:** Average values of clinical and instrumental group P.

	T10m	T20m	T30m	T40m
BIS	72 (42-45)	66 (35-88)	70 (55-82)	74 (52-88)
OAA/S	4 (1-5)	3-4 (1-5)	3-4(1-5)	4 (1-5)

**Table 8:** Average values of clinical and instrumental group M.

	T10m	T20m	T30m	T40m
BIS	64 (48-86)	58 (35-73)	62 (36-84)	66 (48-83)
OAA/S	4 (1-5)	3-4 (1-5)	3-4 (1-5)	4 (1-5)

**Table 9:** Propofol, Midazolam, Remifentanil during MAC.

	Propofol	Midazolam	Remifentanil
onset of sedation	rapid	moderate	rapid
resolution pharmacological effects	rapid	lenta	rapid
injection pain	yes	no	no
intraoperative and postoperative pain	moderate	moderate	minimum
hemodynamic depression	moderate	minimum	minimum
respiratory variations	mild desaturation (<30%)	minimum	moderate
PONV	minimum	minimum	minimum

## **Conclusions**

The combination midazolam-remifentanil presented a lower synergistic effect compared with propofol-remifentanil. The first fact documented a mean BIS of 62.5 +3 vs. 64.7 +4 midazolam-remifentanil association and has finally, although sporadic, incidents of desaturation content and never > 30%. The evaluation of the kinetic values of BIS, the interesting fact that emerges concerns the values> 70, which represented a significant predictor in the study to better recovery of consciousness, which has helped the fast-traking ongoing day-surgery.
